# Creating a German “StadtRaumMonitor” with a climate lens - The approach and design of the piloting

**DOI:** 10.1093/eurpub/ckac129.360

**Published:** 2022-10-25

**Authors:** P Tollmann, O Mekel, M Mensing, T Claßen, T Sammet, R Boos, H Köckler, C Plantz

**Affiliations:** 1 Department Q1, Federal Centre for Health Education, Köln, Germany; 2 NRW Centre for Health, Bochum, Germany; 3 Ministry of Social Affairs, Health and Integration, Stuttgart, Germany; 4 Department of Community Health, University of Applied Sciences, Bochum, Germany

## Abstract

Based on the Scottish Place Standard tool (PST), the Federal Centre for Health Education (BZgA) adapted and piloted a German version called ‘StadtRaumMonitor’ from 2019-2021 as part of a Joint Action ‘Health Equity Europe’ (JAHEE) funded by the EU Commission. The ‘StadtRaumMonitor’ was developed in cooperation with the NRW Centre for Health Nordrhein-Westfalen and the Ministry of Social Affairs, Health and Integration Baden-Württemberg under the leadership of BZgA and with scientific support from the University of Applied Sciences in Bochum. Based on the piloting and the findings of the accompanying scientific research, some adjustments were made to the PST and the website was relaunched offering extended functionality to users. Building on the successes of the ‘StadtRaumMonitor’, a follow-up project started in 2021: Recognising the health impacts of climate change and the need to include climate change issues when assessing the quality of places, the BZgA started a pilot project with the same project partners to adapt the tool to the context of climate change. The “StadtRaumMonitor” with a climate lens aims to address municipal climate adaptation for health promotion. The tool is inspired by the Scottish PST with a climate lens and was developed based on a systematic literature research, focus groups with experts and with actors in municipalities, as well as in participative cooperation with municipal stakeholders. After pre-test and subsequent adjustments, the tool is currently being piloted in four German municipalities, and an evaluation of the application of the tool is taking place. This part of the workshop aims to provide an example of the approach to the further development of an existing tool, here within the context of municipal climate adaptation and on the basis of the “StadtRaumMonitor”, and thereby build competencies in this field. Furthermore, early insights into the piloting of the German PST with a climate lens will be given.

